# Proteomic Profiling of Early Secreted Proteins in Response to Lipopolysaccharide-Induced Vascular Endothelial Cell EA.hy926 Injury

**DOI:** 10.3390/biomedicines11113065

**Published:** 2023-11-15

**Authors:** Worawat Songjang, Nitchawat Paiyabhroma, Noppadon Jumroon, Arunya Jiraviriyakul, Nitirut Nernpermpisooth, Porrnthanate Seenak, Sarawut Kumphune, Siriwan Thaisakun, Narumon Phaonakrop, Sittiruk Roytrakul, Panyupa Pankhong

**Affiliations:** 1 Integrative Biomedical Research Unit (IBRU), Faculty of Allied Health Sciences, Naresuan University, Phitsanulok 65000, Thailand; worawats@nu.ac.th (W.S.);; 2 Department of Medical Technology, Faculty of Allied Health Sciences, Naresuan University, Phitsanulok 65000, Thailand; 3 Department of Cardio-Thoracic Technology, Faculty of Allied Health Sciences, Naresuan University, Phitsanulok 65000, Thailand; 4 Biomedical Engineering and Innovation Research Center, Chiang Mai University, Mueang Chiang Mai District, Chiang Mai 50200, Thailand; 5 Biomedical Engineering Institute (BMEI), Chiang Mai University, Chiang Mai 50200, Thailand; 6 Functional Proteomics Technology Laboratory, National Center for Genetic Engineering and Biotechnology (BIOTEC), National Science and Technology Development Agency, Pathum Thani 12120, Thailand

**Keywords:** sepsis, DAMPs, proteomic, biomarker, lipopolysaccharide, endothelial cell

## Abstract

Sepsis is a crucial public health problem with a high mortality rate caused by a dysregulated host immune response to infection. Vascular endothelial cell injury is an important hallmark of sepsis, which leads to multiple organ failure and death. Early biomarkers to diagnose sepsis may provide early intervention and reduce risk of death. Damage-associated molecular patterns (DAMPs) are host nuclear or cytoplasmic molecules released from cells following tissue damage. We postulated that DAMPs could potentially be a novel sepsis biomarker. We used an in vitro model to determine suitable protein–DAMPs biomarkers for early sepsis diagnosis. Low and high lipopolysaccharide (LPS) doses were used to stimulate the human umbilical vein endothelial cell line EA.hy926 for 24, 48, and 72 h. Results showed that cell viability was reduced in both dose-dependent and time-dependent manners. Cell injury was corroborated by a significant increase in lactate dehydrogenase (LDH) activity within 24 h in cell-conditioned medium. Secreted protein–DAMPs in the supernatant, collected at different time points within 24 h, were characterized using shotgun proteomics LC-MS/MS analysis. Results showed that there were 2233 proteins. Among these, 181 proteins from the LPS-stimulated EA.hy926 at 1, 12, and 24 h were significantly different from those of the control. Twelve proteins were up-regulated at all three time points. Furthermore, a potential interaction analysis of predominant DAMPs-related proteins using STITCH 5.0 revealed the following associations with pathways: response to stress; bacterium; and LPS (GO:0080134; 0009617; 0032496). Markedly, alpha-2-HS-glycoprotein (AHSG or fetuin-A) and lactotransferrin (LTF) potentially presented since the first hour of LPS stimulation, and were highly up-regulated at 24 h. Taken together, we reported proteomic profiling of vascular endothelial cell-specific DAMPs in response to early an in vitro LPS stimulation, suggesting that these early damage-response protein candidates could be novel early biomarkers associated with sepsis.

## 1. Introduction

Sepsis is an important public health problem with a high mortality rate caused by a dysregulated host immune response to infection that causes tissue injury. Globally, sepsis affects approximately 49 million people and results in 11 million deaths every year [1]. It remains the leading cause of death among critically ill patients, with a 30% mortality rate [2]. Gram-negative bacteria are the most common agents that cause sepsis [3]. Gram-negative bacteria’s lipopolysaccharide possesses an endotoxin property that can activate a host’s immune response, which results in endothelial dysfunction, systemic inflammatory response syndrome (SIRS), cell injury, multiple organ failure, and death [4].

Early sepsis diagnosis and prompt management can prevent the risk of septic shock, multiple organ failure, and death. There is no gold standard to definitely diagnose sepsis or predict symptoms’ severity [5]. Mortality prediction for patients with sepsis has been evaluated using the Sequential Organ Failure Assessment (SOFA) scoring system. However, the SOFA system limits the prediction of patient outcomes [6]. Specific biomarkers for both diagnosis and prognosis can aid decision making regarding appropriate treatment. Many biomarkers have emerged (as alternatives to C-reactive protein) for use in sepsis diagnosis and mortality prediction, including interleukin-6 (IL-6), monocyte chemotactic protein 1 (MCP-1), lactate, pentraxin-3, procalcitonin, and presepsin [7,8,9,10,11]. Nevertheless, none of these identified biomarkers are sufficiently specific and sensitive so as to be considered a “gold standard”. Early and specific sepsis biomarkers are urgently required.

Damage-associated molecular patterns (DAMPs) are intracellular molecules released in response to cellular stress, tissue injury, and cell death [12]. Cellular DAMPs include high-mobility group box 1 (HMGB1), histones, heat shock proteins, adenosine triphosphate (ATP), and mitochondrial DNA. DAMPs levels in blood circulation seem to correlate with SIRS/sepsis morbidity and mortality [12,13]. The significance of vascular endothelial-specific DAMPs as diagnostic molecules that could potentially serve as an early biomarker has received limited research attention. In this study, we report the early in vitro identification of vascular endothelial-specific protein–DAMPs in response to lipopolysaccharide (LPS) using the proteomic approach; these findings provide information regarding a novel sepsis biomarker candidate.

## 2. Materials and Methods

### 2.1. Chemical and Reagents

Lipopolysaccharide (LPS) and the Lactate Dehydrogenase Activity Assay Kit were purchased from Sigma (Sigma, St. Louis, MO, USA). Dulbecco’s modified Eagle medium (DMEM), fetal bovine serum, and trypsin–EDTA were purchased from Gibco (Gibco-Thermo Fisher Scientific, New York, NY, USA). The 3-(4,5-dimethyl-2-thiazol)-2,5-diphenyl-2Htetrazolium bromide (MTT) was purchased from Invitrogen (Invitrogen–Thermo Fisher Scientific, USA). All basic chemicals were purchased from Sigma (Sigma, St. Louis, MO, USA).

### 2.2. Cell Type and Cell Culture

Human umbilical vein endothelial cell line (EA.hy926) (ATCC-CRL-2922™) was purchased from American Type Cell Culture (ATCC, Manassas, VA, USA) and cultured in Dulbecco’s modified Eagle medium (DMEM) supplemented with 10% fetal bovine serum (FBS), and 10,000 units of penicillin and streptomycin. Cells were cultured at 37 °C, 5% CO_2_ throughout the experiments.

### 2.3. Determination of Cell Viability

Cell viability was measured using an MTT cell survival assay based on the reduction in MTT (3-[4,5-dimethylthiazol-2-yl]-2,5-diphenyltetrazolium bromide) in the presence of mitochondrial reductases [14]. A 5 × 10^3^/well of cells was seeded in a 96-well plate and incubated overnight. Cells were washed once with PBS before the addition of LPS at final concentrations of 1, 10, and 100 µg/mL to induce cell injury and cell death, and compared with the untreated control at 24, 48, or 72 h. At each time point, the cells were incubated using 0.01 g/mL MTT solution for 3 h at 37 °C. After that, dimethyl sulfoxide (DMSO) was used to dissolve the formazan crystal. Optical density was determined spectrophotometrically at 570 nm. The cell viability percentage was calculated by comparing the optical density of the treated samples with that of the untreated control group (100% viability).

### 2.4. Determination of Released Lactate Dehydrogenase (LDH) Activity

The activity of released lactate dehydrogenase (LDH) was measured to indicate the cell membrane’s permeability loss. LDH activity in the cell culture medium was measured using a Lactate Dehydrogenase Activity Assay Kit (Sigma-Aldrich, St. Louis, MO, USA). Cell culture medium was collected and cell debris was removed using centrifugation. Fifty microliters of triplicate samples were placed in a 96-well plate, followed by fifty microliters of master mix reagent. Mixtures were incubated at 37 °C, and kinetics measured the absorbance at 450 nm every 5 min. LDH activity was calculated using Δ450 and standard curve establishment. 

### 2.5. Determination of Apoptotic Cell Death 

Apoptotic cell death was determined utilizing a Muse™ Cell Analyzer from Millipore (Burlington, MA, USA) following the manufacturer’s instructions. Briefly, after incubation with different LPS concentrations for the indicated time, the treated cells were stained with Annexin V and Dead Cell Reagent (7-AAD) for 20 min before analysis. Results were represented by the events and percentages of live cells, apoptotic cells, and dead cells.

### 2.6. Actin Filaments Immunofluorescent Staining

Cells were seeded into a cell culture slide chamber with a 2.5 × 10^4^/chamber density. After treatment with LPS at the desired concentrations and times, cells were washed with PBS and then fixed with 4% formaldehyde for 15 min. Actin filaments were stained using a Phalloidin-iFluor 555 Reagent (1:100 diluted, ab176756) (Abcam, Cambridge, UK) for 2 h before counter-staining with DAPI for another 20 min. Coverslips were mounted inversely on slides using 95% glycerol, observed under a fluorescence microscope, and photographed.

### 2.7. Sample Preparation for Shotgun Proteomics

The culture medium was transferred to a new tube, mixed well with 2 volumes of cold acetone, and incubated overnight at −20 °C. This mixture was centrifuged at 10,000× *g* for 15 min, and the supernatant was discarded. The pellet was dried and stored at −80 °C prior to use.

### 2.8. In-Solution Trypsin Digestion

Protein concentrations of collected samples were determined via Lowry assay using BSA as a standard protein [15]. Five micrograms of protein samples were subjected to in-solution digestion. Samples were completely dissolved in 10 mM ammonium bicarbonate (AMBIC), disulfide bonds were reduced using 5 mM dithiothreitol (DTT) in 10 mM AMBIC at 60 °C for 1 h, and sulfhydryl groups were alkylated using 15 mM iodoacetamide (IAA) in 10 mM AMBIC at room temperature for 45 min in the dark. Protein samples were digested using sequencing-grade porcine trypsin (1:20 ratio) for 16 h at 37 °C. Tryptic peptides were dried using a speed vacuum concentrator, and then resuspended in 0.1% formic acid for nano-liquid chromatography–tandem mass spectrometry (nano LC-MS/MS) analysis. 

### 2.9. Liquid Chromatography–Tandem Mass Spectrometry (LC-MS/MS)

Tryptic peptide samples were prepared for injection into an Ultimate3000 Nano/Capillary LC System (Thermo Scientific, Oxford, UK) and coupled to a ZenoTOF 7600 mass spectrometer (SCIEX, Framingham, MA, USA). Briefly, one microliter of peptide digests was enriched using a µ-Precolumn 300 µm i.d. × 5 mm C18 Pepmap 100, 5 µm, 100 A (Thermo Scientific, UK), separated using a 75 μm I.D. × 15 cm, and packed with Acclaim PepMap RSLC C18, 2 μm, 100 Å, nanoViper (Thermo Scientific, UK). The C18 column was enclosed in a thermostatted column oven set to 60 °C. Solvents A and B containing 0.1% formic acid in water and 0.1% formic acid in 80% acetonitrile, respectively, were supplied onto the analytical column. A gradient of 5–55% solvent B was used to elute peptides at a constant flow rate of 0.30 μL/min for 30 min. 

The ZenoTOF 7600 system consistently utilized specific source and gas settings throughout all acquisitions. These settings included maintaining ion source gas 1 at 8 psi, curtain gas at 35 psi, CAD gas at 7 psi, a source temperature of 200 °C, positive polarity, and a spray voltage set to 3300 V.

For the DDA method selection, the system chose the top 50 precursor ions with the highest abundance from the survey MS1, considering only those with an intensity exceeding 150 cps. Precursor ions were sampled and experienced dynamic exclusion for 12 s after two instances of MS/MS sampling (with dynamic CE for MS/MS enabling). MS2 spectra were collected in the 100–1800 m/z range with a 50 ms accumulation time, utilizing the Zeno trap. Collision energy equation parameters included an 80 V declustering potential, a 0 V DP spread, and a 0 V CE spread. The system summed time bins to 8 with all channels enabled, employing a Zeno trap threshold of 150,000 cps. The top 60 DDA method used a 3.0 s cycle time.

Regarding bioinformatics and data analysis, MaxQuant 2.2.0.0 was employed to quantify proteins in individual samples using the Andromeda search engine by linking MS/MS spectra to the UniProt Homo sapiens database. Standard settings were applied for label-free quantitation, including a maximum of two missed cleavages and a mass tolerance of 0.6 daltons. The main database search consisted of trypsin digestion, fixing modification via cysteine carbamidomethylation, methionine oxidation, and protein N-terminus acetylation as variable modifications. Only peptides that contained at least seven amino acids and at least one unique peptide were used for protein identification. Identified proteins had at least two peptides, including one unique peptide. Protein FDR was adjusted to 1% and estimated using reversed search sequences. The maximum number of modifications per peptide was limited to 5. The search utilized the Homo sapiens proteome obtained from UniProt as the FASTA file, and potential contaminants were automatically included in the search space from the MaxQuant contaminants.fasta file.

### 2.10. Data Analysis

The MaxQuant ProteinGroups.txt file was loaded into Perseus version 1.6.6.0 [16,17]; potential contaminants that did not correspond to any UPS1 protein were removed from the data set. Maximum intensities were log2 transformed. Missing values were also imputed using Perseus and a constant value (zero). The dataset was normalized, statistically analyzed, and visualized using Metaboanalyst 5.0 [18]. A Venn diagram created using Grapbio 1.0 showing the differences between groups [19]. Predictions of the protein interaction networks were forecasted using STICTH 5.0 [20]. 

### 2.11. Statistical Analysis

Statistical tests were performed using commercially available software (GraphPad Prism version 10.1.0, La Jolla, CA, USA). All values were expressed as mean ± S.E.M. All comparisons were assessed for significance using ANOVA, followed (when appropriate) by the Tukey–Kramer test. A *p*-value less than 0.05 was considered statistically significant.

## 3. Results

### 3.1. LPS-Induced Vascular Endothelial Cell Injury and Cell Death

**Figure 1:**
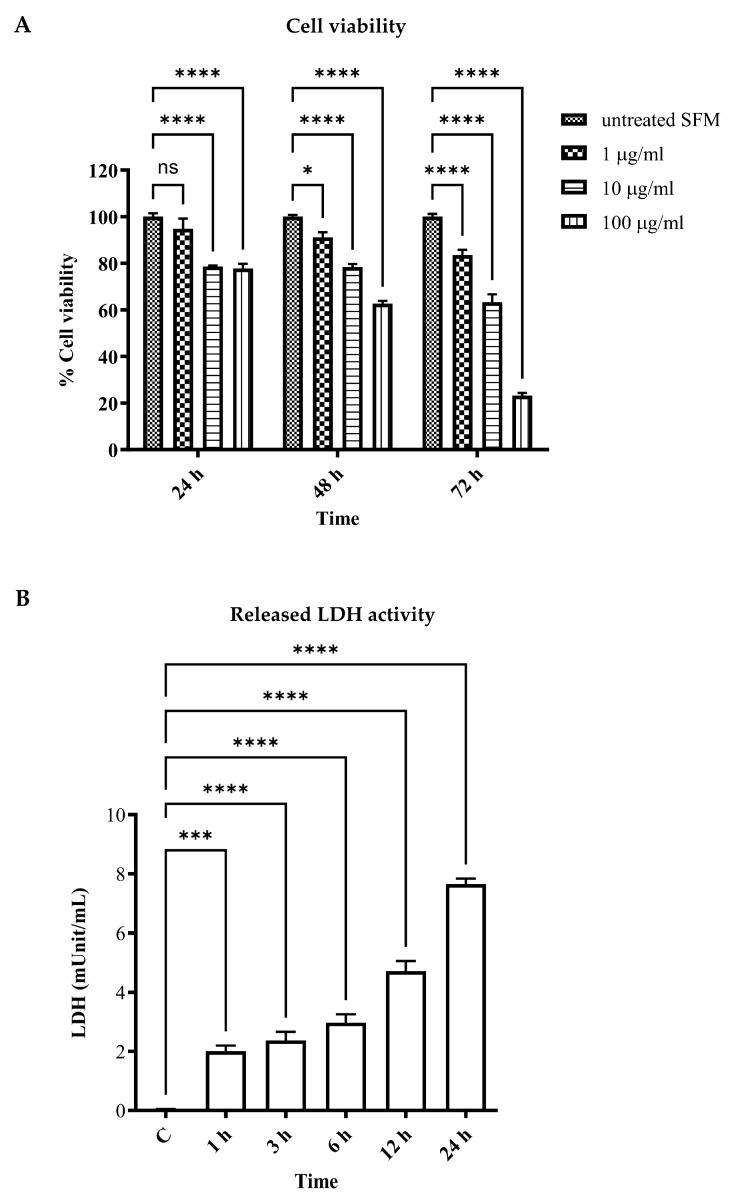
Effects of LPS on human umbilical vein endothelial cell line cell viability and cell injury. EA.hy926 cells were incubated with LPS as per the indicated concentrations and times. Cell viability was assessed using MTT reagents and represented as % cell viability compared to the control (**A**). EA.hy926 cells were incubated with LPS at 1 µg/mL for 1 to 24 h. Cell supernatant was then collected and LDH activity was measured (**B**). * *p* < 0.05, *** *p* = 0.0002, and **** *p* < 0.0001, when compared with the control.

To demonstrate that LPS could induce vascular endothelial cell injury and cell death, which is the first stage of systemic inflammatory response syndrome (SIRS) induction in sepsis, human umbilical vein endothelial cell line EA.hy926 was induced to cell injury and death via LPS for 24–72 h. An MTT assay was performed to determine cell viability. The results showed that the viability of vascular endothelial cells was significantly reduced when stimulated for 24 h using LPS 1, 10, and 100 µg/mL when compared to that of the control (94.75 ± 4.44%, 78.56 ± 0.46%, and 77.75 ± 2.00%, respectively). Also, longer LPS stimulation, for 48 and 72 h, could markedly reduce cell viability in both dose-dependent and time-dependent manners (*p* < 0.05) (91.13 ± 2.18%, 78.37 ± 1.29%, and 62.70 ± 1.14% for LPS 1, 10, and 100 µg/mL at 48 h, respectively; and 83.45 ± 2.35%, 63.26 ± 3.42%, and 23.15 ± 1.20% for LPS 1, 10, and 100 µg/mL at 72 h, respectively) (Figure 1A).

Released lactate dehydrogenase (LDH) activity was performed in LPS-stimulated EA.hy926 for 1, 3, 6, 12, and 24 h. The results showed that released LDH activity was significantly increased in the LPS-stimulated groups in comparison with the control (*p* < 0.05). The longer LPS-stimulated period demonstrated a marked increase in released LDH activity (Figure 1B). These results indicated LPS-induced vascular endothelial cell injury and cell death.

### 3.2. LPS-Altered Actin Cytoskeletal Rearrangement and -Induced Apoptotic Cell Death

**Figure 2:**
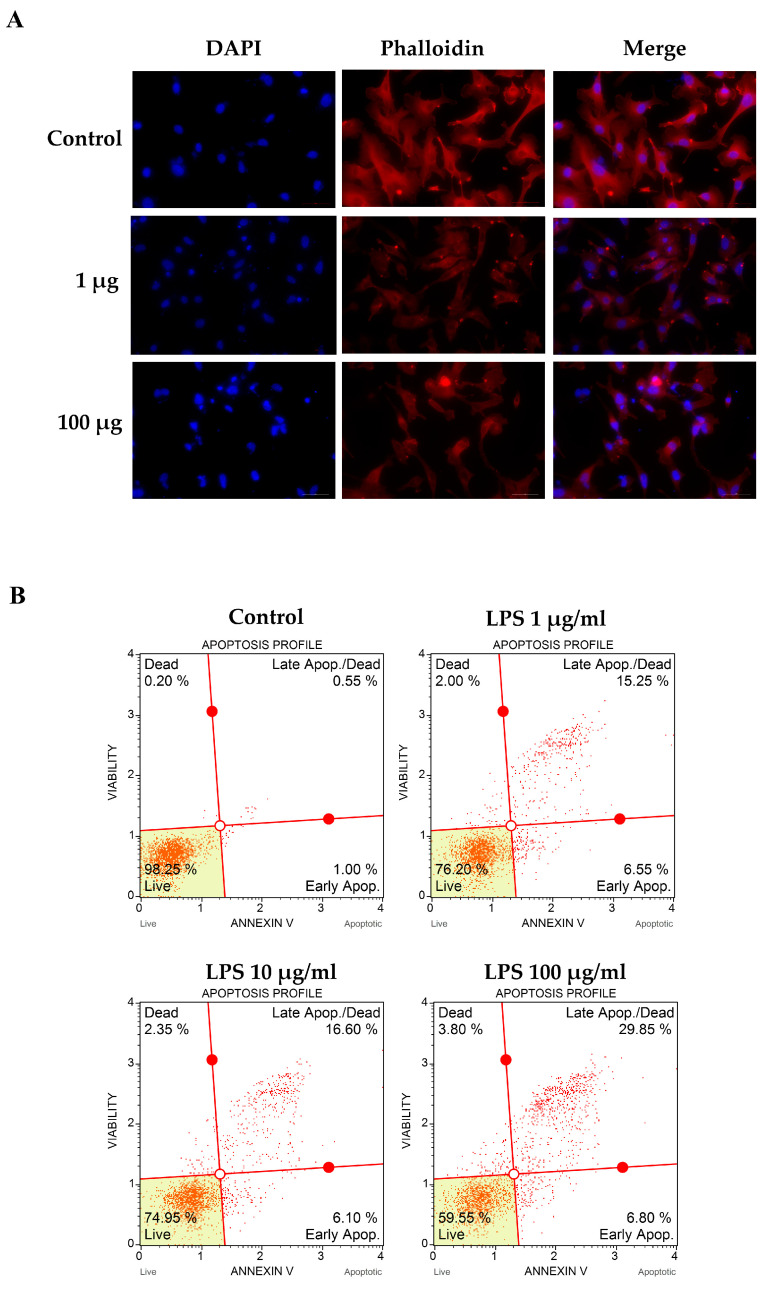
Effects of LPS on filamentous actin arrangement and apoptotic cell death. EA.hy926 cells were incubated with LPS as per the indicated concentrations for 48 h. The treated cell on cell culture slide chamber was stained using fluorescent-tagged phalloidin to visualize the actin filament. Cell images were captured under a fluorescent microscope at 200× magnitude (**A**). Treated cells were stained using Annexin V and Dead Cell Reagent (7-AAD) and analyzed using the Muse™ Cell Analyzer (**B**). Results are represented by the events and percentages of live cells, apoptotic cells, and dead cells.

To determine the effect of LPS on actin rearrangement, cells were treated with LPS for 24 h. Actin filaments were observed via Phalloidin-iFluor 555 reagent staining. The fluorescence microscope showed that LPS-treated cells underwent actin filaments clumping and nuclear condensation when compared to the control. Increasing the LPS concentration could increase the aggregation of actin filaments and pyknotic cells (Figure 2A).

We further determined apoptotic cell death using Annexin V and Dead Cell Reagent (7-AAD). These results showed that apoptotic cell death was increased in LPS-stimulated groups when compared to that of the control (21.8%, 22.7%, and 36.65% for LPS 1, 10, and 100 µg/mL, respectively) (Figure 2B). Taken together, we concluded from these results that LPS could alter actin rearrangement and nuclear condensation, resulting in apoptotic cell death.

### 3.3. Identification of Vascular Endothelial Cell-Specific Protein–DAMPs in Response to LPS

**Figure 3:**
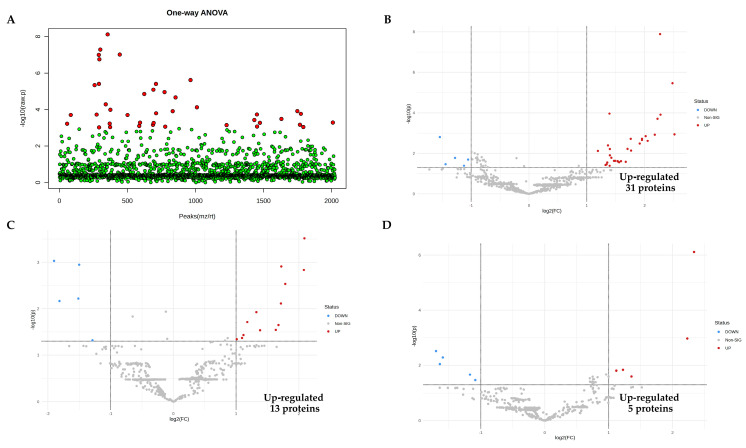

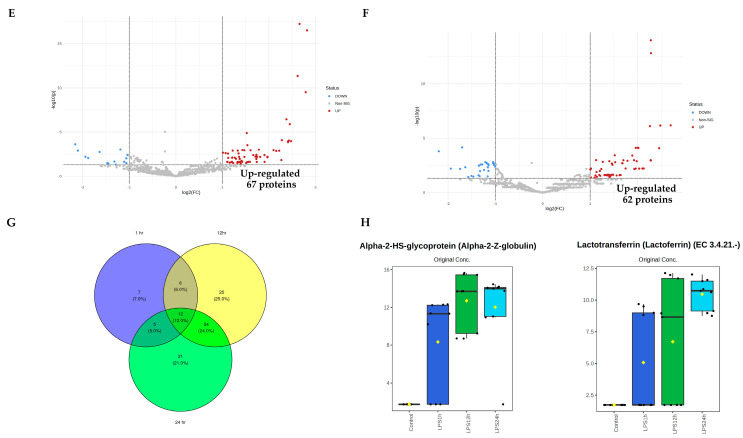
Proteomic analysis identification of proteins in response to LPS. (**A**) The 181 proteins in the LPS-treated groups identified as significantly different (red) from the control group using ANOVA (*p* < 0.05), and no significant differences (green). (**B**–**F**) Volcano plots showing the proteins up-regulated from LPS conditions at 1, 3, 6, 12, and 24 h, respectively. (**G**) Venn diagram demonstrating the interception of up-regulated proteins when induced with LPS at 1, 12, and 24 h. (**H**) The dynamic levels of alpha−2−HS−glycoprotein (AHSG) and lactotransferrin (LTF) increased over time during 1 h, 12 h, and 24 h, respectively.

To identify the expressed proteins related to LPS stimulation, EA.hy926 was induced to cell injury and death via LPS stimulation for 1, 3, 6, 12, and 24 h. At each time point, the treated cell culture media and the control were collected for shotgun proteomic analysis; 2233 differentially expressed proteins (features) across six groups were identified from collected media (Supplementary Materials, Table S1). An analysis of the variance (ANOVA) in the data was performed to analyze the deviation of individual values from the group mean. Fisher’s LSD was performed for all post hoc tests. The results showed that 181 proteins were significantly different from the control (*p* < 0.05), as shown in Figure 3A. 

**Figure 4:**
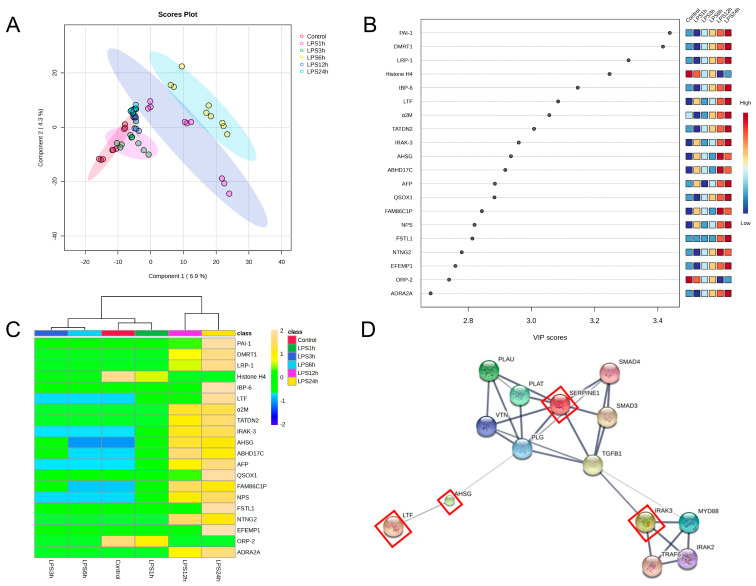
Identification of candidate proteins as novel early sepsis biomarkers. (**A**) The partial least squares-discriminant analysis (PLS-DA) of proteins after LPS stimulation revealed the essential variables of proteins by the eclipse area that differentiate predefined in LPS-treated groups, which are highlighted as 1 h (red), 3 h (green), 6 h (yellow), 12 h (blue) and 24 h (cyan), respectively. (**B**) Variable importance in projection (VIP) score plot and (**C**) heat maps of the top 20 selected proteins. (**D**) STITCH 5.0 analysis of the potential interactions between the 18 candidate proteins (red squares indicate the candidate proteins). PAI-1: plasminogen activator inhibitor 1; DMRT1: doublesex and mab-3-related transcription factor A1; LRP-1: prolow-density lipoprotein receptor-related protein 1; IBP-6: insulin-like growth factor-binding protein 6; LTF: lactotransferrin; α2M: alpha-2-macroglobulin; TATDN2: putative deoxyribonuclease TATDN2; IRAK-3: interleukin-1 receptor-associated kinase 3; AHSG: alpha-2-HS-glycoprotein; ABHD17C: alpha/beta hydrolase domain-containing protein 17C; AFP: alpha-fetoprotein; QSOX1: sulfhydryl oxidase 1; FAM86C1P: putative protein FAM86C1P; NPS: neuropeptide S; FSTL1: follistatin-related protein 1; NTNG2: netrin-G2; EFEMP1: EGF-containing fibulin-like extracellular matrix protein 1; ORP-2: oxysterol-binding protein-related protein 2; and ADRA2A: alpha-2A adrenergic receptor.

A partial least squares-discriminant analysis (PLS-DA) was performed to explain the discrimination of protein alterations between proteomes from LPS-treated groups. The low two-dimensional PLS-DA score plot (PC1 6.9% and PC2 4.3%) suggested differentiation among the group, as shown in Figure 4A. 

### 3.4. Pairwise Comparisons of LPS Conditions

To identify the up-regulated proteins induced by LPS, all five conceivable pairwise comparisons between each LPS condition and the control were performed, in addition to ANOVA, to find differences between each LPS time point. Protein expressions were identified using a volcano plot with at least two fold changes and adjusted significance at *p* < 0.05. In the LPS condition, up-regulated proteins 31, 13, 5, 67, and 62 were significantly statistically higher at 1, 3, 6, 12, and 24 h, respectively, as shown in Figure 3B–F. 

### 3.5. Identification of Candidate Proteins for Novel Early Sepsis Biomarkers

To identify candidate proteins as novel early sepsis biomarkers, 12 proteins from LPS-treated groups were significantly up-regulated at all three time points (1, 12, and 24 h) when compared to the control, as shown in the Venn diagram (Figure 3G); these proteins are listed in Table 1. The dynamic levels of alpha-2-HS-glycoprotein and lactotransferrin are demonstrated in Figure 3H. 

The variable importance in the projection (VIP) plot (Figure 4B) and heat maps (Figure 4C) demonstrated the top 20 proteins that were the most impactful in discriminating between LPS-induced duration and that of the control using the PLS-DA model.

Another strategy that we used to identify LPS-induced vascular endothelial cell-specific proteins as novel early sepsis biomarkers involved considering the 18 up-regulated trend proteins from the VIP plot as biomarker candidates. The STITCH 5.0 interaction potential analysis software was used to predict these 18 proteins. The results revealed that 4 of these 18 proteins (alpha-2-HS-glycoprotein (AHSG), lactotransferrin (LTF), plasminogen activator inhibitor-1 (PAI-1), and interleukin-1 receptor-associated kinase 3 (IRAK-3)) had interactions related to LPS-induced responses, as shown in Figure 4D. Table 2 shows a list of the potential gene ontology (GO) and pathway analysis of the top 18 selected proteins. Both strategies used to investigate early sepsis biomarker candidates identified alpha-2-HS-glycoprotein and lactotransferrin as the dominate proteins in this regard.

## 4. Discussion

This study identified candidate biomarkers by extensively analyzing the proteins released into the medium after various durations of LPS stimulation in human vascular endothelial cells. The 24 h duration of the LPS stimulating period was considered to mimic mild cell injury, which may represent an early sepsis biomarker. To our knowledge, this study provides the first analysis of in vitro LPS-induced secretome in human vascular endothelial cells starting 1 h after stimulation. The well-known in vitro sepsis model was created by stimulating target cells with LPS [21]. We stimulated vascular endothelial cells, which represented the primary sepsis site, with LPS. Therefore, this study’s proteomic profiles were specific to vascular endothelial cells. The proteins that we identified as having been impacted by sepsis and released into blood circulation could be considered as specific markers of early sepsis cell injury.

The severity of LPS-induced endothelial cell injury caused different secretome profiles. At the start, the induction of cell stress and injury might have been mediated by ER stress, in which stress response proteins were mainly secreted through exocytosis via secretory lysosomes and exosomes [22]. Consequently, irreversible compartmental damage led to cell death via either apoptosis or necrosis [23]. In our experiment, we hypothesized that early LPS exposure resulted in cell injury, followed by apoptosis and necrosis, reflected by increments of apoptotic cell percentage and LDH activity in a time-dependent manner. Moreover, ferroptosis, a programmed cell death accompanied by iron accumulation and lipid peroxidation, is directly activated by ROS production [24], and can occur in cell stress conditions. Interestingly, ferroptosis is associated with DAMPs secretion through a passive release mechanism [25]. Therefore, the DAMPs profiled in our study might have been secreted via apoptosis, necrosis, or ferroptosis induction. 

The vascular endothelial cell-specific proteins secreted from cells in response to LPS could also be considered as protein damage-associated molecular patterns (DAMPs). A suitable sepsis biomarker requires rapid release into blood circulation following sepsis, and to remain at a detectable level in the blood circulation. In this study, we found 12 proteins that were significantly up-regulated at 1, 12, and 24 h after LPS-stimulation when compared to the control. Alpha-2-HS-glycoprotein (AHSG) and lactotransferrin (LTF) showed a tendency to increase in a time-dependent manner. In addition, 18 up-regulated-trend proteins were found to be impacted by sepsis using a VIP plot based on PLS-DA results. Four of these eighteen proteins (AHSG, LTF, PAI-1, and IRAK-3) showed a strong relation to LPS-induced responses. 

It is known that the induction of inflammation on endothelial cells increases proinflammatory cytokine production. Similarly, our data showed that some cytokines, chemokines, and their related receptors were up-regulated in the conditioned medium (for example, interleukin-12 (IL-12), C-X-C motif chemokine ligand 5 (CXCL5), tumor necrosis factor ligand superfamily member 12 (TNFSF12), and TNF receptor-associated factor 2 (TRAF2)), especially after 12–24 h of LPS stimulation (Supplementary Materials, Table S1). However, there was an absence of an expression of common cytokines, such as IL-1β, IL-6, and TNF-α. Previous studies have identified LPS-induced intracellular cytokine production using RT-qPCR and immunoblotting; however, for proteomic analysis of secretomes, these cytokines were not shown as predominant proteins [26,27].

Kwon et al. [27] identified the protein moesin as a sepsis biomarker. This protein was identified in supernatant collected from LPS-stimulated endothelial cells at 24 h. The proteomic approach was performed based on two-dimensional gel electrophoresis. Comparing Kwon et al.’s research and the present investigation revealed that no proteins were identified in common, except a small amount of alpha-2-HS-glycoprotein, which was secreted at 24 h in Kwon et al.’s report. This might be due to the different study design and proteomic approach.

Two candidate proteins, alpha-2-HS-glycoprotein (AHSG) and lactotransferrin (LFT), were sustainably found from the first hour until 24 h. Lactotransferrin (LTF), or lactoferrin, is a multifunctional protein present in diverse biological secretions, including milk, tears, saliva, vaginal fluids, and sperm. It has a broad range of functions, including iron binding/transfer, as well as antibacterial, antiviral, antifungal, anti-inflammatory, and anticarcinogenic properties [28]. However, a role of LTF in sepsis has also recently been reported. Tong et al. [29] identified five differentially expressed proteins in the peripheral blood mononuclear cells (PBMCs) of sepsis patients via proteomic analysis. These five proteins, including LTF, HMGB1, matrix metalloproteinase 8 (MMP8), neutrophil gelatinase-associated lipocalin (NGAL), and grancalcin (GCA), were proposed as candidate diagnostic biomarkers for sepsis. Our findings demonstrating that LTF secreted from vascular endothelial cells 1 h after LPS stimulation emphasize the early specificity of this protein for use as a biomarker for sepsis diagnosis.

Alpha-2-HS-glycoprotein (AHSG), or fetuin-A, is an abundant glycoprotein circulating in human plasma. It is primarily secreted by the liver, but many other cells can produce this protein [30]. Its synthesis is divergently regulated in response to injury or infection, including sepsis, classifying it as a negative or positive acute-phase protein [31]. A recent study reported the roles of the negative acute-phase protein fetuin-A, and the positive acute-phase protein presepsin, for neonatal sepsis diagnosis. Significantly increased presepsin levels were associated with sepsis, whereas fetuin-A levels were similar to those of the control [32]. However, our data showed fetuin-A’s role as a positive acute-phase protein during early sepsis when compared with the control. These controversial data require further investigation.

A recent review article demonstrated several emerging sepsis biomarkers that have been used in the emergency room, including presepsin, soluble triggering receptor expressed on myeloid cells-1 (sTREM-1), and proadrenomedullin [33]. These emerging biomarkers elicit more sensitivity and specificity than CRP and procalcitonin do. However, more clinical trials are required to evaluate their performance. Furthermore, a single protein may not provide sufficient sensitivity and specificity for use as a sepsis diagnostic biomarker. Pilar-Orive et al. [34] used a panel of three proteins (serum amyloid-A1 (SAA-1), soluble interleukin-2 alpha chain receptor (sCD25), and leucine-rich alpha-2 glycoprotein (LRG1)) as a sepsis diagnosis marker for children. They found that this three-protein panel was able to distinguish patients with sepsis from healthy subjects. Therefore, promising early biomarkers for sepsis diagnosis may need to include a panel of proteins instead of a single protein.

This study had some limitations. Firstly, the vascular endothelial cell line (EA.hy926) used in this study may not reflect the real physiology of the endothelial in vitro model. Primary vascular endothelial cells, such as HUVECs, are considered ideal models for studying the biology of vascular endothelial cells. However, there are some considerations for using a primary cell culture in this study. HUVECs have a limited life span when cultured in a laboratory setting, and their observable characteristics may undergo alterations throughout subsequent passages. Therefore, the immortalized endothelial cell line EA.hy926, which demonstrates endothelial cell characteristics, including angiogenesis, homeostasis/thrombosis, blood pressure regulation, and inflammation [35,36], was selected as a model for vascular endothelial cells to provide proof-of-concept information. Secondly, this study identified the candidate proteins as potential DAMPs due to their natural release from the injured endothelial cells. Previous reports have indicated DAMPs’ function in innate immune cell activation via PRRs [37,38]. However, this study’s findings might not provide DAMPs’ molecular mechanism and function regarding sepsis. Further studies are required to investigate the physiological roles of candidate DAMPs and their mechanisms related to sepsis, including a clinical sample of sepsis patients to validate the efficacy of the measurement methods and protein markers, that could be applied in clinical settings.

In conclusion, this study used an in vitro sepsis model to provide proof-of-concept information. This study’s findings provide a compilation of early in vitro protein–DAMPs specifically released in response to LPS-induced vascular endothelial cell injury. Additionally, this study identified many noteworthy proteins that have the potential to serve as novel early biomarkers, particularly AHSG and LTF, due to their consistent presence in response to LPS stimulation.

## Figures and Tables

**Table 1:** List of the 12 candidate proteins that were up-regulated at 1, 12, and 24 h after LPS stimulation.

Protein Names	f. Value	*p*. Value	−log10p	FDR
Transforming growth factor beta-3 (TGF-beta-3)	26.734	7.57 × 10^−9^	8.121	1.53 × 10^−5^
Alpha-2-HS-glycoprotein (alpha-2-Z-globulin)	21.088	1.00 × 10^−7^	6.9986	5.08 × 10^−5^
Lactotransferrin (lactoferrin) (EC 3.4.21.-)	19.989	1.75 × 10^−7^	6.7575	7.08 × 10^−5^
Albumin	14.497	3.89 × 10^−6^	5.4101	0.000988
GTP-binding protein Di-Ras3 (distinct subgroup of the Ras family member 3)	14.25	4.54 × 10^−6^	5.3428	0.001022
Transforming growth factor beta activator LRRC32 (garpin) (leucine-rich repeat domain-containing protein 32)	12.867	1.11 × 10^−5^	4.955	0.002043
Hexokinase HKDC1 (EC 2.7.1.1) (hexokinase domain-containing protein 1)	11.876	2.17 × 10^−5^	4.6632	0.003384
Zinc finger protein 865	10.661	5.15 × 10^−5^	4.2884	0.007448
PRKCA-binding protein (protein kinase C-alpha-binding protein)	9.5197	0.000121	3.9175	0.013742
Integrin alpha-1	7.6929	0.000524	3.281	0.037017
Sororin (cell division cycle-associated protein 5) (p35)	7.6665	0.000535	3.2713	0.037017
Ankyrin repeat domain-containing protein 36B (CLL-associated antigen KW-1)	7.3272	0.000714	3.1466	0.041303

**Table 2:** List of potential gene ontology (GO) and pathway analysis of the top 18 selected proteins.

Pathway ID	Pathway Description	False Discovery Rate
GO:0080134	regulation of response to stress	4.40 × 10^−9^
GO:0051707	response to other organism	7.76 × 10^−8^
GO:0009617	response to bacterium	8.73 × 10^−8^
GO:0031347	regulation of defense response	8.73 × 10^−8^
GO:0048583	regulation of response to stimuli	1.37 × 10^−7^
GO:0032496	response to lipopolysaccharide	5.06 × 10^−6^

## Data Availability

Data presented in this study are available on request from the corresponding author.

## Supplementary Materials

The following supporting information can be downloaded at: https://www.mdpi.com/article/10.3390/biomedicines11113065/s1, Table S1: Table of protein names and proteomic profiling intensity.

## References

[1] World Health Organization Sepsis. https://www.who.int/news-room/fact-sheets/detail/sepsis.

[2] Rhodes A., Evans L.E., Alhazzani W., Levy M.M., Antonelli M., Ferrer R., Kumar A., Sevransky J.E., Sprung C.L., Nunnally M.E. (2017). Surviving Sepsis Campaign: International Guidelines for Management of Sepsis and Septic Shock: 2016. Intensive Care Med..

[3] Chun K., Syndergaard C., Damas C., Trubey R., Mukindaraj A., Qian S., Jin X., Breslow S., Niemz A. (2015). Sepsis Pathogen Identification. J. Lab. Autom..

[4] Dauphinee S.M., Karsan A. (2006). Lipopolysaccharide signaling in endothelial cells. Lab. Investing..

[5] Cohen J., Vincent J.L., Adhikari N.K., Machado F.R., Angus D.C., Calandra T., Jaton K., Giulieri S., Delaloye J., Opal S. (2015). Sepsis: A roadmap for future research. Lancet. Infect. Dis..

[6] Singer M., Deutschman C.S., Seymour C.W., Shankar-Hari M., Annane D., Bauer M., Bellomo R., Bernard G.R., Chiche J.D., Coopersmith C.M. (2016). The Third International Consensus Definitions for Sepsis and Septic Shock (Sepsis-3). JAMA.

[7] Barre M., Behnes M., Hamed S., Pauly D., Lepiorz D., Lang S., Akin I., Borggrefe M., Bertsch T., Hoffmann U. (2018). Revisiting the prognostic value of monocyte chemotactic protein 1 and interleukin-6 in the sepsis-3 era. J. Crit. Care.

[8] Behnes M., Bertsch T., Lepiorz D., Lang S., Trinkmann F., Brueckmann M., Borggrefe M., Hoffmann U. (2014). Diagnostic and prognostic utility of soluble CD 14 subtype (presepsin) for severe sepsis and septic shock during the first week of intensive care treatment. Crit. Care.

[9] Liu S., Wang X., She F., Zhang W., Liu H., Zhao X. (2021). Effects of Neutrophil-to-Lymphocyte Ratio Combined with Interleukin-6 in Predicting 28-Day Mortality in Patients with Sepsis. Front. Immunol..

[10] Song J., Moon S., Park D.W., Cho H.J., Kim J.Y., Park J., Cha J.H. (2020). Biomarker combination and SOFA score for the prediction of mortality in sepsis and septic shock: A prospective observational study according to the Sepsis-3 definitions. Medicine.

[11] De Nooijer A.H., Pickkers P., Netea M.G., Kox M. (2023). Inflammatory biomarkers to predict the prognosis of acute bacterial and viral infections. J. Crit. Care.

[12] Zhou M., Aziz M., Wang P. (2021). Damage-Associated Molecular Patterns as Double-Edged Swords in Sepsis. Antioxid. Redox Signal..

[13] Itagaki K., Rica I., Konecna B., Kim H.I., Park J., Kaczmarek E., Hauser C.J. (2021). Role of Mitochondria-Derived Danger Signals Released after Injury in Systemic Inflammation and Sepsis. Antioxid. Redox Signal..

[14] Kongpol K., Nernpermpisooth N., Prompunt E., Kumphune S. (2019). Endothelial-Cell-Derived Human Secretory Leukocyte Protease Inhibitor (SLPI) Protects Cardiomyocytes against Ischemia/Reperfusion Injury. Biomolecules.

[15] Lowry O.H., Rosebrough N.J., Farr A.L., Randall R.J. (1951). Protein measurement with the Folin phenol reagent. J. Biol. Chem..

[16] Tyanova S., Temu T., Cox J. (2016). The MaxQuant computational platform for mass spectrometry-based shotgun proteomics. Nat. Protoc..

[17] Tyanova S., Temu T., Sinitcyn P., Carlson A., Hein M.Y., Geiger T., Mann M., Cox J. (2016). The Perseus computational platform for comprehensive analysis of (prote)omics data. Nat. Methods.

[18] Pang Z., Zhou G., Ewald J., Chang L., Hacariz O., Basu N., Xia J. (2022). Using MetaboAnalyst 5.0 for LC–HRMS spectra processing, multi-omics integration and covariate adjustment of global metabolomics data. Nat. Protoc..

[19] Zhao T., Wang Z. (2022). GraphBio: A shiny web app to easily perform popular visualization analysis for omics data. Front. Genet..

[20] Mi H., Muruganujan A., Ebert D., Huang X., Thomas P.D. (2019). PANTHER version 14: More genomes, a new PANTHER GO-slim and improvements in enrichment analysis tools. Nucleic Acids Res..

[21] Quoilin C., Mouithys-Mickalad A., Lécart S., Fontaine-Aupart M.P., Hoebeke M. (2014). Evidence of oxidative stress and mitochondrial respiratory chain dysfunction in an in vitro model of sepsis-induced kidney injury. Biochim. Biophys. Acta (BBA)-Bioenerg..

[22] Li W., Deng M., Loughran P.A., Yang M., Lin M., Yang C., Gao W., Jin S., Li S., Cai J. (2020). LPS Induces Active HMGB1 Release from Hepatocytes into Exosomes through the Coordinated Activities of TLR4 and Caspase-11/GSDMD Signaling. Front. Immunol..

[23] Murao A., Aziz M., Wang H., Brenner M., Wang P. (2021). Release mechanisms of major DAMPs. Apoptosis.

[24] Tang D., Chen X., Kang R., Kroemer G. (2021). Ferroptosis: Molecular mechanisms and health implications. Cell Res..

[25] Wen Q., Liu J., Kang R., Zhou B., Tang D. (2019). The release and activity of HMGB1 in ferroptosis. Biochem. Biophys. Res. Commun..

[26] Hossain K.H., Okamoto T., Usuda H., Jahan I., Niibayashi T., Wada K. (2020). Differential Expression of Pro-inflammatory and Pro-coagulant Genes in Endothelial Cells Induced by *Porphyromonas gingivalis* Lipopolysaccharide, *Escherichia coli* Lipopolysaccharide, and Zymosan. Shimane J. Med. Sci..

[27] Kwon O.K., Lee W., Kim S.J., Lee Y.M., Lee J.Y., Kim J.Y., Bae J.S., Lee S. (2015). In-depth proteomics approach of secretome to identify novel biomarker for sepsis in LPS-stimulated endothelial cells. Electrophoresis.

[28] Wang B., Timilsena Y.P., Blanch E., Adhikari B. (2019). Lactoferrin: Structure, function, denaturation and digestion. Crit. Rev. Food Sci. Nutr..

[29] Tong Y., Ku X., Wu C., Liu J., Yang C., Tang W., Yan W., Tang J. (2019). Data-independent acquisition-based quantitative proteomic analysis reveals differences in host immune response of peripheral blood mononuclear cells to sepsis. Scand. J. Immunol..

[30] Lin Y.H., Zhu J., Meijer S., Franc V., Heck A.J.R. (2019). Glycoproteogenomics: A frequent gene polymorphism affects the glycosylation pattern of the human serum fetuin/alpha-2-HS-glycoprotein. Mol. Cell. Proteom. MCP.

[31] Wang H., Sama A.E. (2012). Anti-inflammatory role of fetuin-A in injury and infection. Curr. Mol. Med..

[32] Degirmencioglu H., Ozer Bekmez B., Derme T., Oncel M.Y., Canpolat F.E., Tayman C. (2019). Presepsin and fetuin-A dyad for the diagnosis of proven sepsis in preterm neonates. BMC Infect. Dis..

[33] Turgman O., Schinkel M., Wiersinga W.J. (2023). Host response biomarkers for sepsis in the emergency room. Crit. Care.

[34] Pilar-Orive F.J.A.I., Azkargorta M., Elortza F., Garcia-Obregon S. (2022). A three-protein panel to support the diagnosis of sepsis in children. J. Clin. Med..

[35] Baranska P., Jerczynska H., Pawlowska Z., Koziolkiewicz W., Cierniewski C.S. (2005). Expression of Integrins and Adhesive Properties of Human Endothelial Cell Line EA.hy 926. Cancer Genom. Proteom..

[36] Bauer J., Margolis M., Schreiner C., Edgell C.J., Azizkhan J., Lazarowski E., Juliano R.L. (1992). In vitro model of angiogenesis using a human endothelium-derived permanent cell line: Contributions of induced gene expression, G-proteins, and integrins. J. Cell. Physiol..

[37] Siqueiros-Cendón T., Arévalo-Gallegos S., Iglesias-Figueroa B.F., García-Montoya I.A., Salazar-Martínez J., Rascón-Cruz Q. (2014). Immunomodulatory effects of lactoferrin. Acta Pharmacol. Sin..

[38] Kan C., Yang J., Fan H., Dai Y., Wang X., Chen R., Liu J., Meng X., Wang W., Li G. (2022). Fetuin-A is an immunomodulator and a potential therapeutic option in BMP4-dependent heterotopic ossification and associated bone mass loss. Bone Res..

